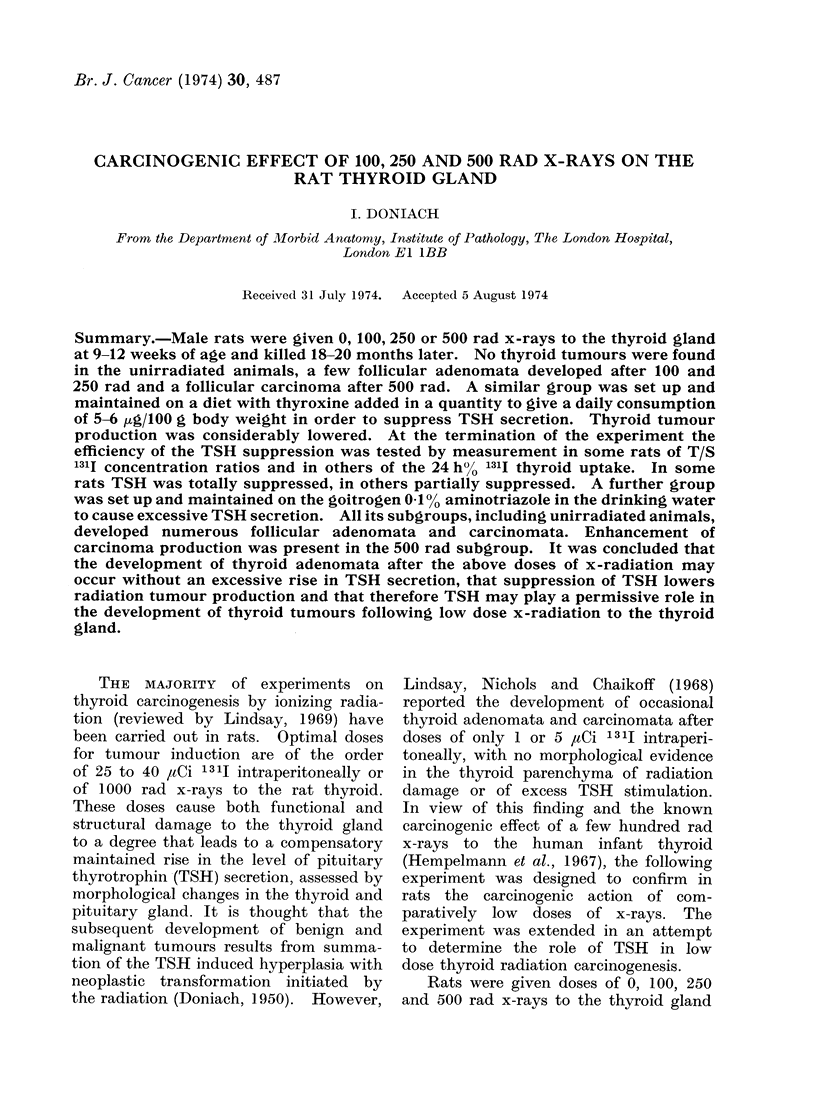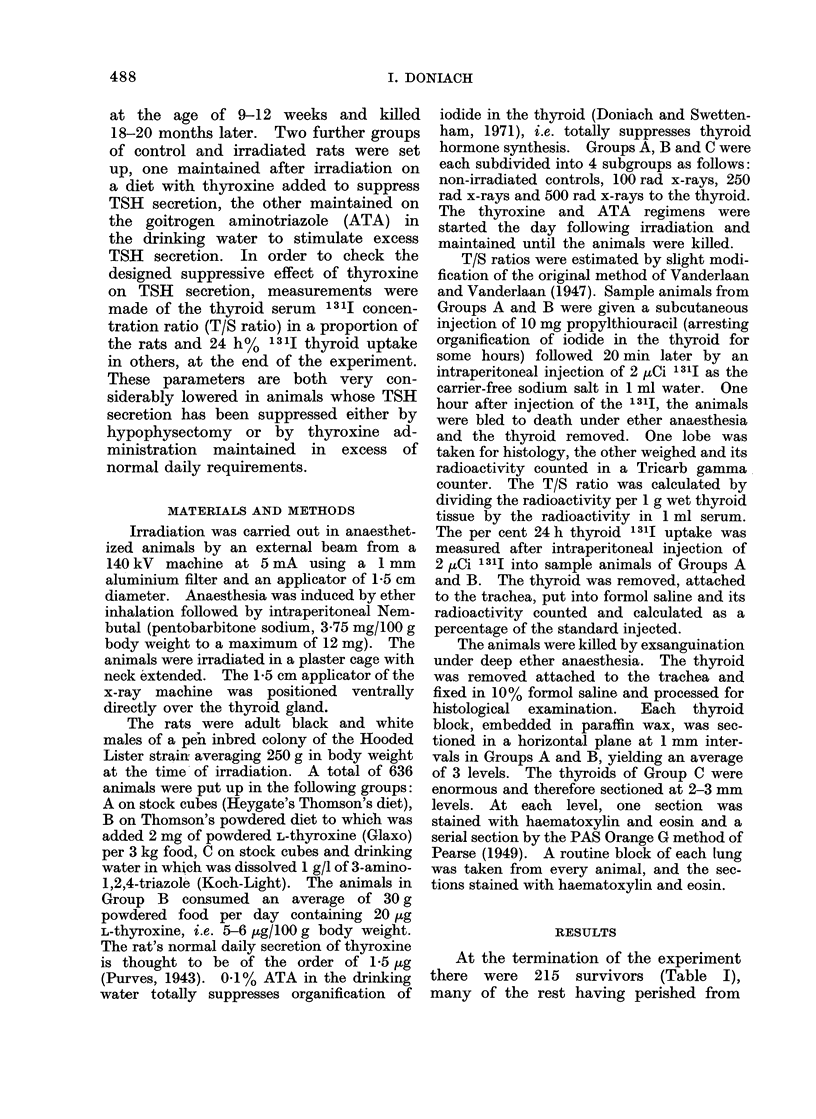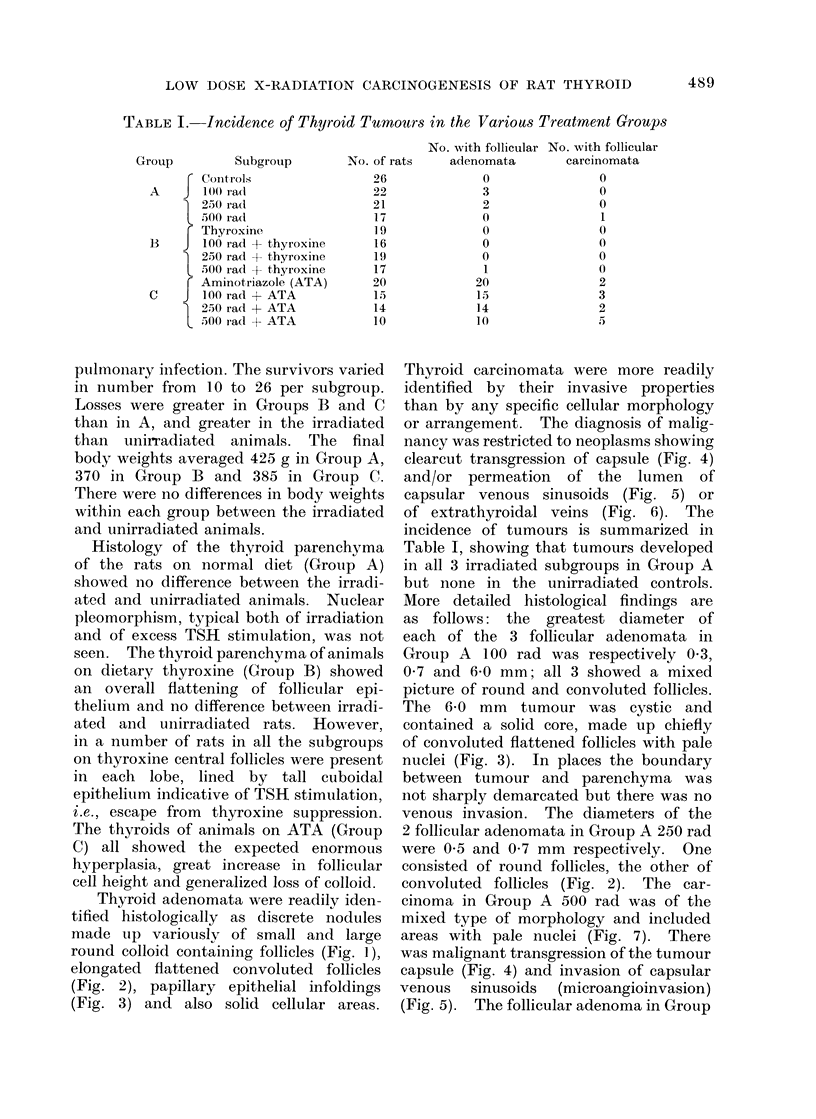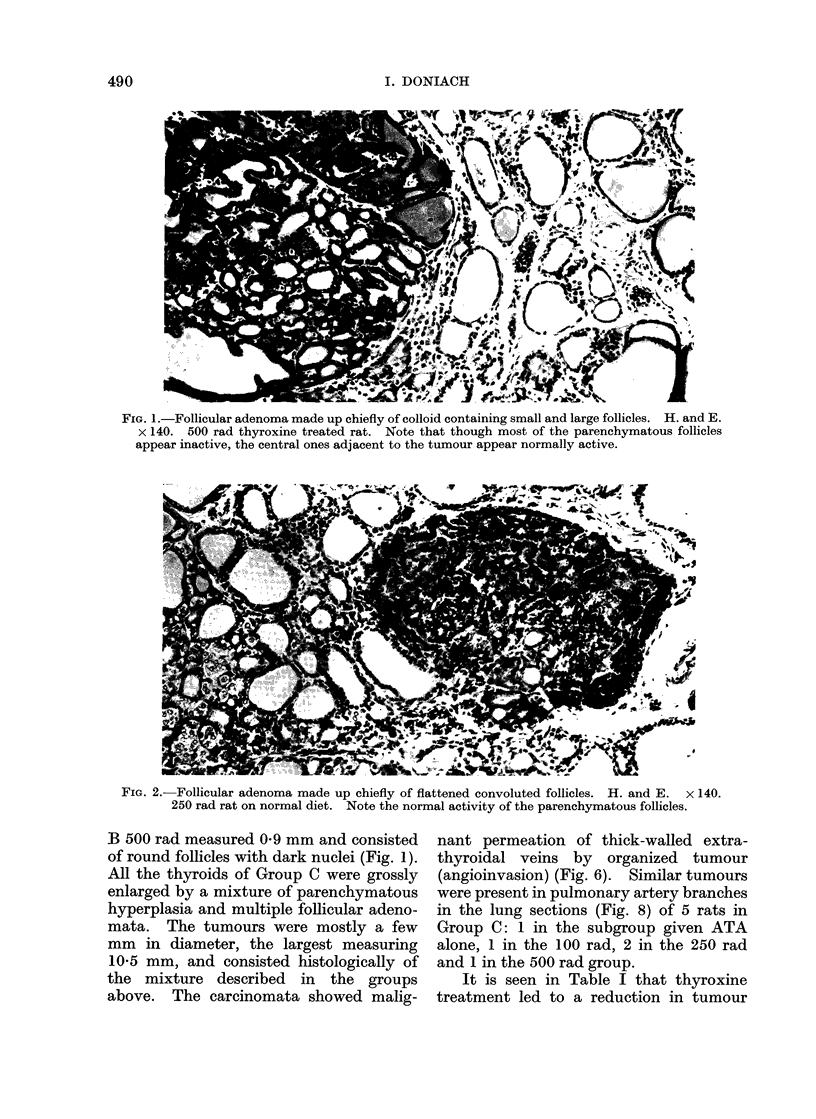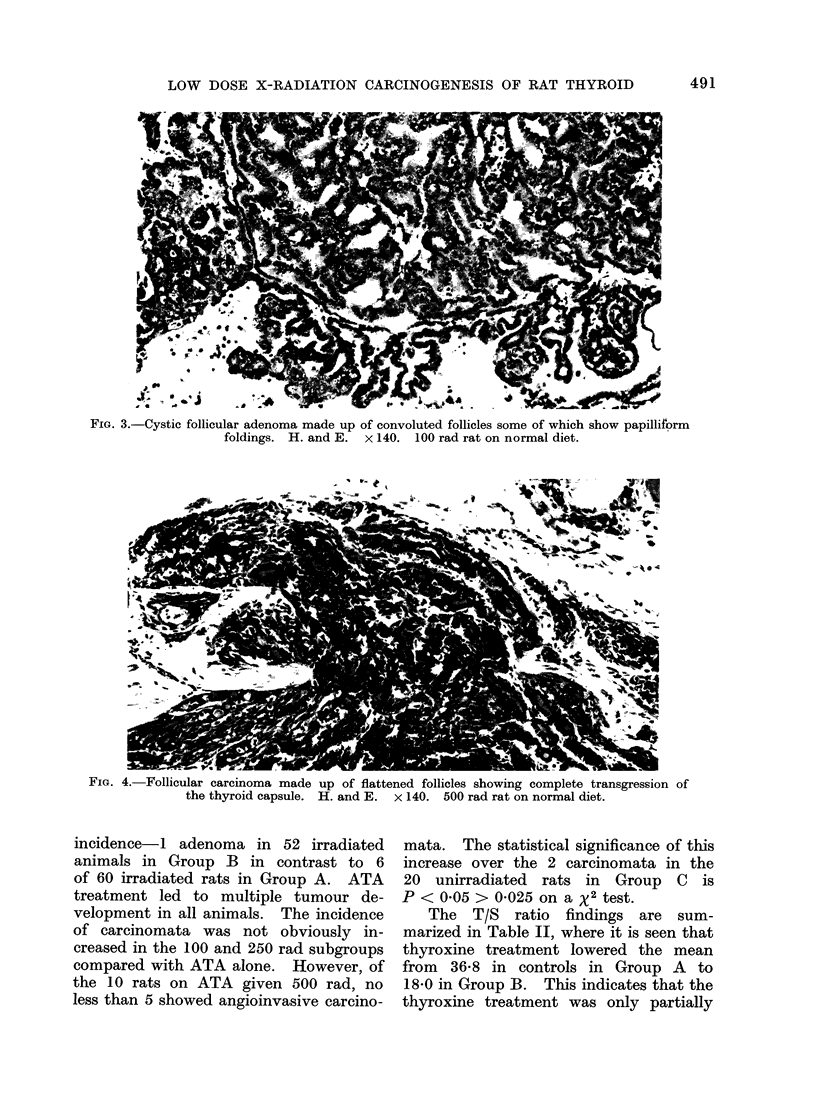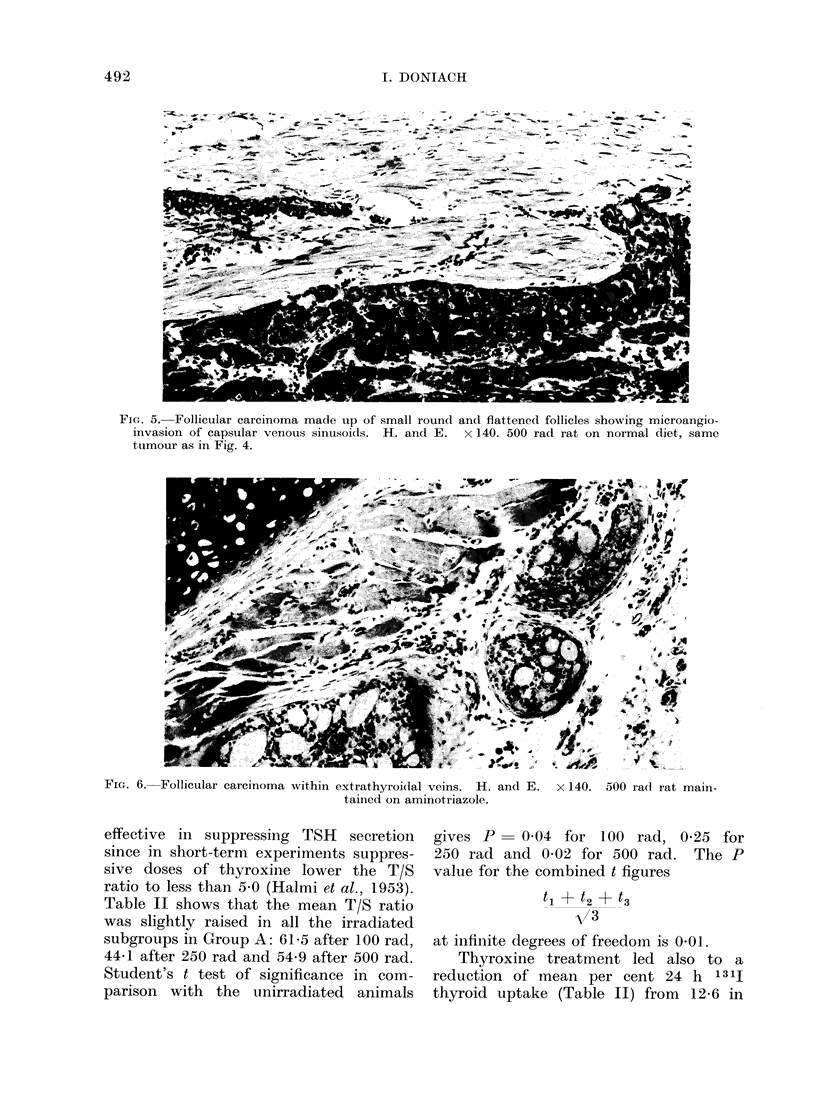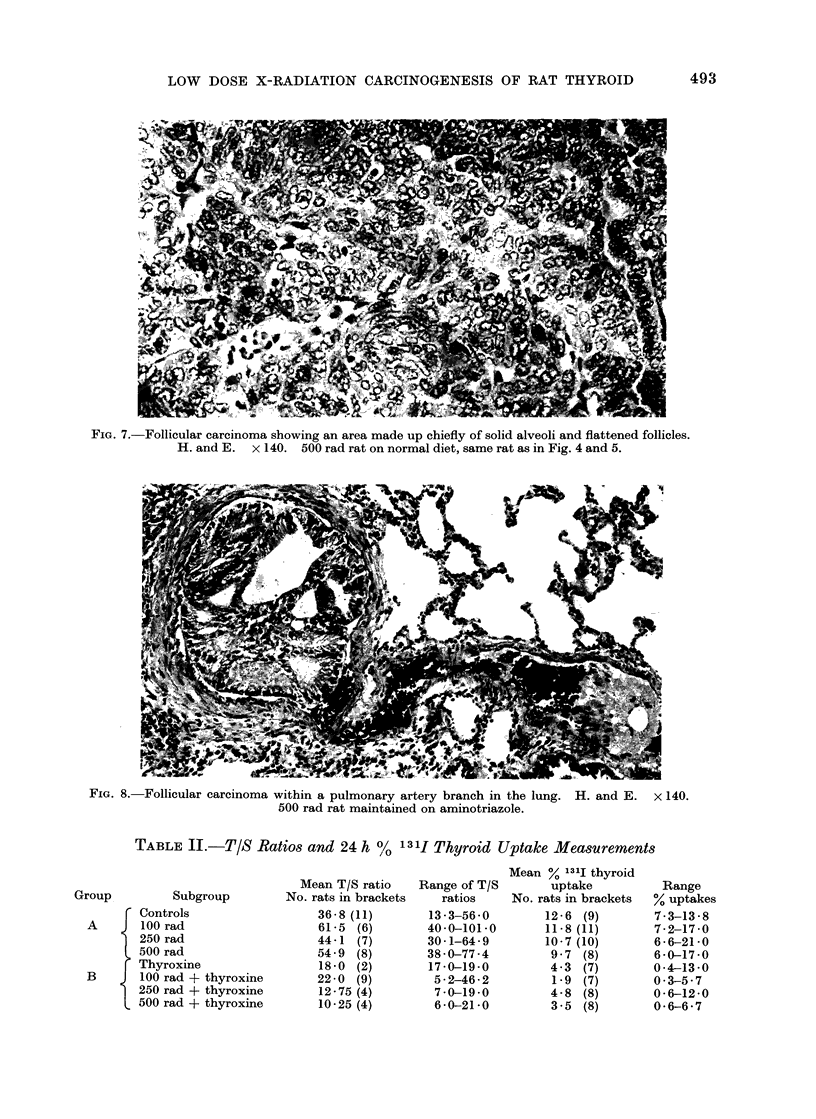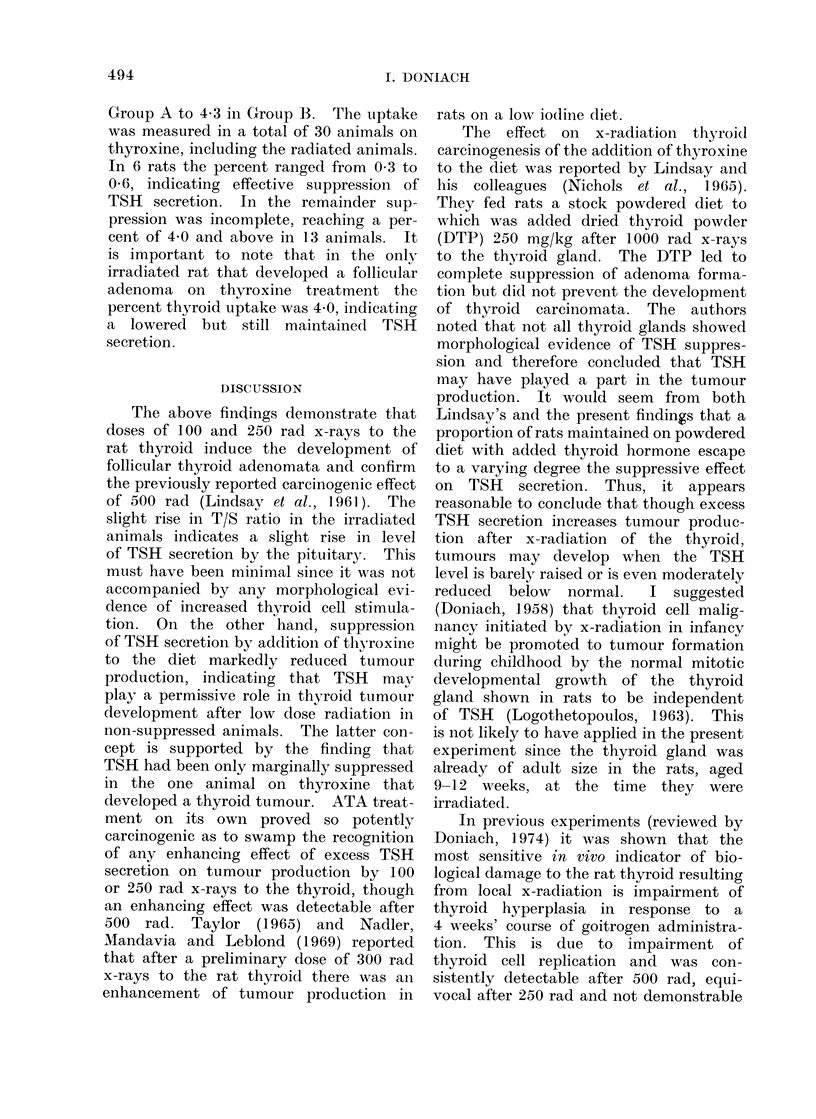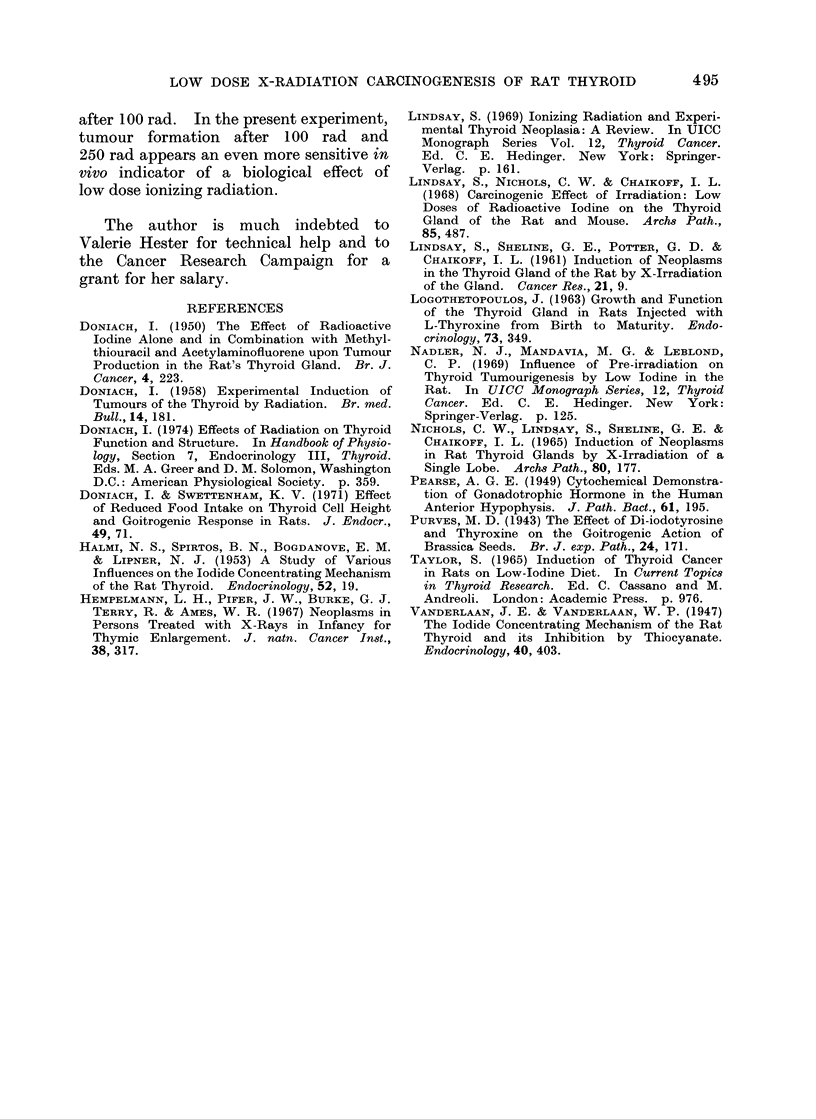# Carcinogenic Effect of 100, 250 and 500 Rad X-Rays on the Rat Thyroid Gland

**DOI:** 10.1038/bjc.1974.227

**Published:** 1974-12

**Authors:** I. Doniach

## Abstract

**Images:**


*Br. J. Cancer* (1974) **30**, 487

### CARCINOGENIC EFFECT OF 100, 250 AND 500 RAD X-RAYS ON THE RAT THYROID GLAND

I. DONIACH

*From the Department of Morbid Anatomy, Institute of Pathology, The London Hospital, London E1 1BB*

Received 31 July 1974. Accepted 5 August 1974

**Summary.—Male rats were given 0, 100, 250 or 500 rad x-rays to the thyroid gland at 9–12 weeks of age and killed 18–20 months later. No thyroid tumours were found in the unirradiated animals, a few follicular adenomata developed after 100 and 250 rad and a follicular carcinoma after 500 rad. A similar group was set up and maintained on a diet with thyroxine added in a quantity to give a daily consumption of 5–6 $\mu$g/100 g body weight in order to suppress TSH secretion. Thyroid tumour production was considerably lowered. At the termination of the experiment the efficiency of the TSH suppression was tested by measurement in some rats of T/S $^{131}$I concentration ratios and in others of the 24 h% $^{131}$I thyroid uptake. In some rats TSH was totally suppressed, in others partially suppressed. A further group was set up and maintained on the goitrogen 0·1% aminotriazole in the drinking water to cause excessive TSH secretion. All its subgroups, including unirradiated animals, developed numerous follicular adenomata and carcinomata. Enhancement of carcinoma production was present in the 500 rad subgroup. It was concluded that the development of thyroid adenomata after the above doses of x-radiation may occur without an excessive rise in TSH secretion, that suppression of TSH lowers radiation tumour production and that therefore TSH may play a permissive role in the development of thyroid tumours following low dose x-radiation to the thyroid gland.**

THE MAJORITY of experiments on thyroid carcinogenesis by ionizing radiation (reviewed by Lindsay, 1969) have been carried out in rats. Optimal doses for tumour induction are of the order of 25 to 40 $\mu$Ci $^{131}$I intraperitoneally or of 1000 rad x-rays to the rat thyroid. These doses cause both functional and structural damage to the thyroid gland to a degree that leads to a compensatory maintained rise in the level of pituitary thyrotrophin (TSH) secretion, assessed by morphological changes in the thyroid and pituitary gland. It is thought that the subsequent development of benign and malignant tumours results from summation of the TSH induced hyperplasia with neoplastic transformation initiated by the radiation (Doniach, 1950). However, Lindsay, Nichols and Chaikoff (1968) reported the development of occasional thyroid adenomata and carcinomata after doses of only 1 or 5 $\mu$Ci $^{131}$I intraperitoneally, with no morphological evidence in the thyroid parenchyma of radiation damage or of excess TSH stimulation. In view of this finding and the known carcinogenic effect of a few hundred rad x-rays to the human infant thyroid (Hempelmann *et al.*, 1967), the following experiment was designed to confirm in rats the carcinogenic action of comparatively low doses of x-rays. The experiment was extended in an attempt to determine the role of TSH in low dose thyroid radiation carcinogenesis.

Rats were given doses of 0, 100, 250 and 500 rad x-rays to the thyroid gland at the age of 9–12 weeks and killed 18–20 months later. Two further groups of control and irradiated rats were set up, one maintained after irradiation on a diet with thyroxine added to suppress TSH secretion, the other maintained on the goitrogen aminotriazole (ATA) in the drinking water to stimulate excess TSH secretion. In order to check the designed suppressive effect of thyroxine on TSH secretion, measurements were made of the thyroid serum $^{131}$I concentration ratio (T/S ratio) in a proportion of the rats and 24 h% $^{131}$I thyroid uptake in others, at the end of the experiment. These parameters are both very considerably lowered in animals whose TSH secretion has been suppressed either by hypophysectomy or by thyroxine administration maintained in excess of normal daily requirements.

### MATERIALS AND METHODS

Irradiation was carried out in anaesthetized animals by an external beam from a 140 kV machine at 5 mA using a 1 mm aluminium filter and an applicator of 1·5 cm diameter. Anaesthesia was induced by ether inhalation followed by intraperitoneal Nembutal (pentobarbitone sodium, 3·75 mg/100 g body weight to a maximum of 12 mg). The animals were irradiated in a plaster cage with neck extended. The 1·5 cm applicator of the x-ray machine was positioned ventrally directly over the thyroid gland.

The rats were adult black and white males of a pen inbred colony of the Hooded Lister strain averaging 250 g in body weight at the time of irradiation. A total of 636 animals were put up in the following groups: A on stock cubes (Heygate's Thomson's diet), B on Thomson's powdered diet to which was added 2 mg of powdered L-thyroxine (Glaxo) per 3 kg food, C on stock cubes and drinking water in which was dissolved 1 g/l of 3-amino-1,2,4-triazole (Koch-Light). The animals in Group B consumed an average of 30 g powdered food per day containing 20 $\mu$g L-thyroxine, *i.e.* 5–6 $\mu$g/100 g body weight. The rat's normal daily secretion of thyroxine is thought to be of the order of 1·5 $\mu$g (Purves, 1943). 0·1% ATA in the drinking water totally suppresses organification of iodide in the thyroid (Doniach and Swettenham, 1971), *i.e.* totally suppresses thyroid hormone synthesis. Groups A, B and C were each subdivided into 4 subgroups as follows: non-irradiated controls, 100 rad x-rays, 250 rad x-rays and 500 rad x-rays to the thyroid. The thyroxine and ATA regimens were started the day following irradiation and maintained until the animals were killed.

T/S ratios were estimated by slight modification of the original method of Vanderlaan and Vanderlaan (1947). Sample animals from Groups A and B were given a subcutaneous injection of 10 mg propylthiouracil (arresting organification of iodide in the thyroid for some hours) followed 20 min later by an intraperitoneal injection of 2 $\mu$Ci $^{131}$I as the carrier-free sodium salt in 1 ml water. One hour after injection of the $^{131}$I, the animals were bled to death under ether anaesthesia and the thyroid removed. One lobe was taken for histology, the other weighed and its radioactivity counted in a Tricarb gamma counter. The T/S ratio was calculated by dividing the radioactivity per 1 g wet thyroid tissue by the radioactivity in 1 ml serum. The per cent 24 h thyroid $^{131}$I uptake was measured after intraperitoneal injection of 2 $\mu$Ci $^{131}$I into sample animals of Groups A and B. The thyroid was removed, attached to the trachea, put into formol saline and its radioactivity counted and calculated as a percentage of the standard injected.

The animals were killed by exsanguination under deep ether anaesthesia. The thyroid was removed attached to the trachea and fixed in 10% formol saline and processed for histological examination. Each thyroid block, embedded in paraffin wax, was sectioned in a horizontal plane at 1 mm intervals in Groups A and B, yielding an average of 3 levels. The thyroids of Group C were enormous and therefore sectioned at 2–3 mm levels. At each level, one section was stained with haematoxylin and eosin and a serial section by the PAS Orange G method of Pearse (1949). A routine block of each lung was taken from every animal, and the sections stained with haematoxylin and eosin.

### RESULTS

At the termination of the experiment there were 215 survivors (Table I), many of the rest having perished from

TABLE I.—*Incidence of Thyroid Tumours in the Various Treatment Groups*

| Group | Subgroup | No. of rats | No. with follicular adenomata | No. with follicular carcinomata |
|---|---|---|---|---|
| A | Controls | 26 | 0 | 0 |
| | 100 rad | 22 | 3 | 0 |
| | 250 rad | 21 | 2 | 0 |
| | 500 rad | 17 | 0 | 1 |
| B | Thyroxine | 19 | 0 | 0 |
| | 100 rad + thyroxine | 16 | 0 | 0 |
| | 250 rad + thyroxine | 19 | 0 | 0 |
| | 500 rad + thyroxine | 17 | 1 | 0 |
| C | Aminotriazole (ATA) | 20 | 20 | 2 |
| | 100 rad + ATA | 15 | 15 | 3 |
| | 250 rad + ATA | 14 | 14 | 2 |
| | 500 rad + ATA | 10 | 10 | 5 |

pulmonary infection. The survivors varied in number from 10 to 26 per subgroup. Losses were greater in Groups B and C than in A, and greater in the irradiated than unirradiated animals. The final body weights averaged 425 g in Group A, 370 in Group B and 385 in Group C. There were no differences in body weights within each group between the irradiated and unirradiated animals.

Histology of the thyroid parenchyma of the rats on normal diet (Group A) showed no difference between the irradiated and unirradiated animals. Nuclear pleomorphism, typical both of irradiation and of excess TSH stimulation, was not seen. The thyroid parenchyma of animals on dietary thyroxine (Group B) showed an overall flattening of follicular epithelium and no difference between irradiated and unirradiated rats. However, in a number of rats in all the subgroups on thyroxine central follicles were present in each lobe, lined by tall cuboidal epithelium indicative of TSH stimulation, *i.e.*, escape from thyroxine suppression. The thyroids of animals on ATA (Group C) all showed the expected enormous hyperplasia, great increase in follicular cell height and generalized loss of colloid.

Thyroid adenomata were readily identified histologically as discrete nodules made up variously of small and large round colloid containing follicles (Fig. 1), elongated flattened convoluted follicles (Fig. 2), papillary epithelial infoldings (Fig. 3) and also solid cellular areas. Thyroid carcinomata were more readily identified by their invasive properties than by any specific cellular morphology or arrangement. The diagnosis of malignancy was restricted to neoplasms showing clearcut transgression of capsule (Fig. 4) and/or permeation of the lumen of capsular venous sinusoids (Fig. 5) or of extrathyroidal veins (Fig. 6). The incidence of tumours is summarized in Table I, showing that tumours developed in all 3 irradiated subgroups in Group A but none in the unirradiated controls. More detailed histological findings are as follows: the greatest diameter of each of the 3 follicular adenomata in Group A 100 rad was respectively 0·3, 0·7 and 6·0 mm; all 3 showed a mixed picture of round and convoluted follicles. The 6·0 mm tumour was cystic and contained a solid core, made up chiefly of convoluted flattened follicles with pale nuclei (Fig. 3). In places the boundary between tumour and parenchyma was not sharply demarcated but there was no venous invasion. The diameters of the 2 follicular adenomata in Group A 250 rad were 0·5 and 0·7 mm respectively. One consisted of round follicles, the other of convoluted follicles (Fig. 2). The carcinoma in Group A 500 rad was of the mixed type of morphology and included areas with pale nuclei (Fig. 7). There was malignant transgression of the tumour capsule (Fig. 4) and invasion of capsular venous sinusoids (microangioinvasion) (Fig. 5). The follicular adenoma in Group

FIG. 1.—Follicular adenoma made up chiefly of colloid containing small and large follicles. H. and E. ×140. 500 rad thyroxine treated rat. Note that though most of the parenchymatous follicles appear inactive, the central ones adjacent to the tumour appear normally active.

FIG. 2.—Follicular adenoma made up chiefly of flattened convoluted follicles. H. and E. ×140. 250 rad rat on normal diet. Note the normal activity of the parenchymatous follicles.

B 500 rad measured 0·9 mm and consisted of round follicles with dark nuclei (Fig. 1). All the thyroids of Group C were grossly enlarged by a mixture of parenchymatous hyperplasia and multiple follicular adenomata. The tumours were mostly a few mm in diameter, the largest measuring 10·5 mm, and consisted histologically of the mixture described in the groups above. The carcinomata showed malignant permeation of thick-walled extrathyroidal veins by organized tumour (angioinvasion) (Fig. 6). Similar tumours were present in pulmonary artery branches in the lung sections (Fig. 8) of 5 rats in Group C: 1 in the subgroup given ATA alone, 1 in the 100 rad, 2 in the 250 rad and 1 in the 500 rad group.

It is seen in Table I that thyroxine treatment led to a reduction in tumour

FIG. 3.—Cystic follicular adenoma made up of convoluted follicles some of which show papilliform foldings. H. and E. ×140. 100 rad rat on normal diet.

FIG. 4.—Follicular carcinoma made up of flattened follicles showing complete transgression of the thyroid capsule. H. and E. ×140. 500 rad rat on normal diet.

incidence—1 adenoma in 52 irradiated animals in Group B in contrast to 6 of 60 irradiated rats in Group A. ATA treatment led to multiple tumour development in all animals. The incidence of carcinomata was not obviously increased in the 100 and 250 rad subgroups compared with ATA alone. However, of the 10 rats on ATA given 500 rad, no less than 5 showed angioinvasive carcinomata. The statistical significance of this increase over the 2 carcinomata in the 20 unirradiated rats in Group C is $P < 0{\cdot}05 > 0{\cdot}025$ on a $\chi^2$ test.

The T/S ratio findings are summarized in Table II, where it is seen that thyroxine treatment lowered the mean from 36·8 in controls in Group A to 18·0 in Group B. This indicates that the thyroxine treatment was only partially

FIG. 5.—Follicular carcinoma made up of small round and flattened follicles showing microangio-invasion of capsular venous sinusoids. H. and E. ×140. 500 rad rat on normal diet, same tumour as in Fig. 4.

FIG. 6.—Follicular carcinoma within extrathyroidal veins. H. and E. ×140. 500 rad rat maintained on aminotriazole.

effective in suppressing TSH secretion since in short-term experiments suppressive doses of thyroxine lower the T/S ratio to less than 5·0 (Halmi *et al.*, 1953). Table II shows that the mean T/S ratio was slightly raised in all the irradiated subgroups in Group A: 61·5 after 100 rad, 44·1 after 250 rad and 54·9 after 500 rad. Student's $t$ test of significance in comparison with the unirradiated animals gives $P = 0{\cdot}04$ for 100 rad, 0·25 for 250 rad and 0·02 for 500 rad. The $P$ value for the combined $t$ figures

$$\frac{t_1 + t_2 + t_3}{\sqrt{3}}$$

at infinite degrees of freedom is 0·01.

Thyroxine treatment led also to a reduction of mean per cent 24 h $^{131}$I thyroid uptake (Table II) from 12·6 in

FIG. 7.—Follicular carcinoma showing an area made up chiefly of solid alveoli and flattened follicles. H. and E. ×140. 500 rad rat on normal diet, same rat as in Fig. 4 and 5.

FIG. 8.—Follicular carcinoma within a pulmonary artery branch in the lung. H. and E. ×140. 500 rad rat maintained on aminotriazole.

TABLE II.—*T/S Ratios and 24 h % $^{131}I$ Thyroid Uptake Measurements*

| Group | Subgroup | Mean T/S ratio No. rats in brackets | Range of T/S ratios | Mean % $^{131}$I thyroid uptake No. rats in brackets | Range % uptakes |
|---|---|---|---|---|---|
| A | Controls | 36·8 (11) | 13·3–56·0 | 12·6 (9) | 7·3–13·8 |
| | 100 rad | 61·5 (6) | 40·0–101·0 | 11·8 (11) | 7·2–17·0 |
| | 250 rad | 44·1 (7) | 30·1–64·9 | 10·7 (10) | 6·6–21·0 |
| | 500 rad | 54·9 (8) | 38·0–77·4 | 9·7 (8) | 6·0–17·0 |
| B | Thyroxine | 18·0 (2) | 17·0–19·0 | 4·3 (7) | 0·4–13·0 |
| | 100 rad + thyroxine | 22·0 (9) | 5·2–46·2 | 1·9 (7) | 0·3–5·7 |
| | 250 rad + thyroxine | 12·75 (4) | 7·0–19·0 | 4·8 (8) | 0·6–12·0 |
| | 500 rad + thyroxine | 10·25 (4) | 6·0–21·0 | 3·5 (8) | 0·6–6·7 |

Group A to 4·3 in Group B. The uptake was measured in a total of 30 animals on thyroxine, including the radiated animals. In 6 rats the percent ranged from 0·3 to 0·6, indicating effective suppression of TSH secretion. In the remainder suppression was incomplete, reaching a percent of 4·0 and above in 13 animals. It is important to note that in the only irradiated rat that developed a follicular adenoma on thyroxine treatment the percent thyroid uptake was 4·0, indicating a lowered but still maintained TSH secretion.

### DISCUSSION

The above findings demonstrate that doses of 100 and 250 rad x-rays to the rat thyroid induce the development of follicular thyroid adenomata and confirm the previously reported carcinogenic effect of 500 rad (Lindsay *et al.*, 1961). The slight rise in T/S ratio in the irradiated animals indicates a slight rise in level of TSH secretion by the pituitary. This must have been minimal since it was not accompanied by any morphological evidence of increased thyroid cell stimulation. On the other hand, suppression of TSH secretion by addition of thyroxine to the diet markedly reduced tumour production, indicating that TSH may play a permissive role in thyroid tumour development after low dose radiation in non-suppressed animals. The latter concept is supported by the finding that TSH had been only marginally suppressed in the one animal on thyroxine that developed a thyroid tumour. ATA treatment on its own proved so potently carcinogenic as to swamp the recognition of any enhancing effect of excess TSH secretion on tumour production by 100 or 250 rad x-rays to the thyroid, though an enhancing effect was detectable after 500 rad. Taylor (1965) and Nadler, Mandavia and Leblond (1969) reported that after a preliminary dose of 300 rad x-rays to the rat thyroid there was an enhancement of tumour production in rats on a low iodine diet.

The effect on x-radiation thyroid carcinogenesis of the addition of thyroxine to the diet was reported by Lindsay and his colleagues (Nichols *et al.*, 1965). They fed rats a stock powdered diet to which was added dried thyroid powder (DTP) 250 mg/kg after 1000 rad x-rays to the thyroid gland. The DTP led to complete suppression of adenoma formation but did not prevent the development of thyroid carcinomata. The authors noted that not all thyroid glands showed morphological evidence of TSH suppression and therefore concluded that TSH may have played a part in the tumour production. It would seem from both Lindsay's and the present findings that a proportion of rats maintained on powdered diet with added thyroid hormone escape to a varying degree the suppressive effect on TSH secretion. Thus, it appears reasonable to conclude that though excess TSH secretion increases tumour production after x-radiation of the thyroid, tumours may develop when the TSH level is barely raised or is even moderately reduced below normal. I suggested (Doniach, 1958) that thyroid cell malignancy initiated by x-radiation in infancy might be promoted to tumour formation during childhood by the normal mitotic developmental growth of the thyroid gland shown in rats to be independent of TSH (Logothetopoulos, 1963). This is not likely to have applied in the present experiment since the thyroid gland was already of adult size in the rats, aged 9–12 weeks, at the time they were irradiated.

In previous experiments (reviewed by Doniach, 1974) it was shown that the most sensitive *in vivo* indicator of biological damage to the rat thyroid resulting from local x-radiation is impairment of thyroid hyperplasia in response to a 4 weeks' course of goitrogen administration. This is due to impairment of thyroid cell replication and was consistently detectable after 500 rad, equivocal after 250 rad and not demonstrable after 100 rad. In the present experiment, tumour formation after 100 rad and 250 rad appears an even more sensitive *in vivo* indicator of a biological effect of low dose ionizing radiation.

The author is much indebted to Valerie Hester for technical help and to the Cancer Research Campaign for a grant for her salary.

### REFERENCES

Doniach, I. (1950) The Effect of Radioactive Iodine Alone and in Combination with Methylthiouracil and Acetylaminofluorene upon Tumour Production in the Rat's Thyroid Gland. *Br. J. Cancer*, **4**, 223.

Doniach, I. (1958) Experimental Induction of Tumours of the Thyroid by Radiation. *Br. med. Bull.*, **14**, 181.

Doniach, I. (1974) Effects of Radiation on Thyroid Function and Structure. In *Handbook of Physiology*, Section 7, Endocrinology III, *Thyroid*. Eds. M. A. Greer and D. M. Solomon, Washington D.C.: American Physiological Society. p. 359.

Doniach, I. & Swettenham, K. V. (1971) Effect of Reduced Food Intake on Thyroid Cell Height and Goitrogenic Response in Rats. *J. Endocr.*, **49**, 71.

Halmi, N. S., Spirtos, B. N., Bogdanove, E. M. & Lipner, N. J. (1953) A Study of Various Influences on the Iodide Concentrating Mechanism of the Rat Thyroid. *Endocrinology*, **52**, 19.

Hempelmann, L. H., Pifer, J. W., Burke, G. J. Terry, R. & Ames, W. R. (1967) Neoplasms in Persons Treated with X-Rays in Infancy for Thymic Enlargement. *J. natn. Cancer Inst.*, **38**, 317.

Lindsay, S. (1969) Ionizing Radiation and Experimental Thyroid Neoplasia: A Review. In UICC Monograph Series Vol. 12, *Thyroid Cancer*. Ed. C. E. Hedinger. New York: Springer-Verlag. p. 161.

Lindsay, S., Nichols, C. W. & Chaikoff, I. L. (1968) Carcinogenic Effect of Irradiation: Low Doses of Radioactive Iodine on the Thyroid Gland of the Rat and Mouse. *Archs Path.*, **85**, 487.

Lindsay, S., Sheline, G. E., Potter, G. D. & Chaikoff, I. L. (1961) Induction of Neoplasms in the Thyroid Gland of the Rat by X-Irradiation of the Gland. *Cancer Res.*, **21**, 9.

Logothetopoulos, J. (1963) Growth and Function of the Thyroid Gland in Rats Injected with L-Thyroxine from Birth to Maturity. *Endocrinology*, **73**, 349.

Nadler, N. J., Mandavia, M. G. & Leblond, C. P. (1969) Influence of Pre-irradiation on Thyroid Tumourigenesis by Low Iodine in the Rat. In *UICC Monograph Series*, 12, *Thyroid Cancer*. Ed. C. E. Hedinger. New York: Springer-Verlag. p. 125.

Nichols, C. W., Lindsay, S., Sheline, G. E. & Chaikoff, I. L. (1965) Induction of Neoplasms in Rat Thyroid Glands by X-Irradiation of a Single Lobe. *Archs Path.*, **80**, 177.

Pearse, A. G. E. (1949) Cytochemical Demonstration of Gonadotrophic Hormone in the Human Anterior Hypophysis. *J. Path. Bact.*, **61**, 195.

Purves, M. D. (1943) The Effect of Di-iodotyrosine and Thyroxine on the Goitrogenic Action of Brassica Seeds. *Br. J. exp. Path.*, **24**, 171.

Taylor, S. (1965) Induction of Thyroid Cancer in Rats on Low-Iodine Diet. In *Current Topics in Thyroid Research*. Ed. C. Cassano and M. Andreoli. London: Academic Press. p. 976.

Vanderlaan, J. E. & Vanderlaan, W. P. (1947) The Iodide Concentrating Mechanism of the Rat Thyroid and its Inhibition by Thiocyanate. *Endocrinology*, **40**, 403.